# Enhanced production of bacterial cellulose by using a biofilm reactor and its material property analysis

**DOI:** 10.1186/1754-1611-3-12

**Published:** 2009-07-24

**Authors:** Kuan-Chen Cheng, Jeff M Catchmark, Ali Demirci

**Affiliations:** 1 Department of Agricultural and Biological Engineering, The Pennsylvania State University, University Park, Pennsylvania 16802, USA; 2 The Huck Institutes of Life Sciences, The Pennsylvania State University University Park, Pennsylvania 16802, USA

## Abstract

Bacterial cellulose has been used in the food industry for applications such as low-calorie desserts, salads, and fabricated foods. It has also been used in the paper manufacturing industry to enhance paper strength, the electronics industry in acoustic diaphragms for audio speakers, the pharmaceutical industry as filtration membranes, and in the medical field as wound dressing and artificial skin material. In this study, different types of plastic composite support (PCS) were implemented separately within a fermentation medium in order to enhance bacterial cellulose (BC) production by *Acetobacter xylinum*. The optimal composition of nutritious compounds in PCS was chosen based on the amount of BC produced. The selected PCS was implemented within a bioreactor to examine the effects on BC production in a batch fermentation. The produced BC was analyzed using X-ray diffraction (XRD), field emission scanning electron microscopy (FESEM), thermogravimetric analysis (TGA), and dynamic mechanical analysis (DMA). Among thirteen types of PCS, the type SFYR+ was selected as solid support for BC production by *A. xylinum *in a batch biofilm reactor due to its high nitrogen content, moderate nitrogen leaching rate, and sufficient biomass attached on PCS. The PCS biofilm reactor yielded BC production (7.05 g/L) that was 2.5-fold greater than the control (2.82 g/L). The XRD results indicated that the PCS-grown BC exhibited higher crystallinity (93%) and similar crystal size (5.2 nm) to the control. FESEM results showed the attachment of *A. xylinum *on PCS, producing an interweaving BC product. TGA results demonstrated that PCS-grown BC had about 95% water retention ability, which was lower than BC produced within suspended-cell reactor. PCS-grown BC also exhibited higher *T*_max _compared to the control. Finally, DMA results showed that BC from the PCS biofilm reactor increased its mechanical property values, i.e., stress at break and Young's modulus when compared to the control BC. The results clearly demonstrated that implementation of PCS within agitated fermentation enhanced BC production and improved its mechanical properties and thermal stability.

## Introduction

Cellulose is the most abundant macromolecule on earth [1] and most cellulose is produced by vascular plants. A substitute to reduce the demand from plants is the production of cellulose using a microbial system [1,2]. Bacterial cellulose (BC) has been used in the food industry for applications such as low-calorie desserts, salads, and fabricated food, in the paper manufacturing industry to enhance paper strength, in acoustic diaphragms for audio speakers, and in the pharmaceutical industry as a filtration membrane, wound dressing and artificial skin [3,4].

BC produced by *Acetobacter xylinum *in static cultures is initially extruded from the cell surface as microfibers and entangle together to form ribbons, which then intertwine to form a dense, gelatinous pellicle at the air/liquid interface. Traditional static culture has been used for BC production, which produces pellicles on the surface of fermentation broth. The pellicle grows downward since cells that are entrapped into the pellicle become inactive or die from lack of oxygen [5].

Several cultivation improvements have been presented to enhance BC production; Yoshino et al. [6] developed a cylindrical silicone membrane vessel that provided oxygen from the bottom, resulting in a two-fold improvement in BC production. Serafica et al. [7] made bacterial cellulose in a rotating disk bioreactor that consists of a cylindrical trough with inoculated medium into which are dipped flat, circular disks mounted on a rotating central shaft. A rotating disk bioreactor is more efficient and reduces the time of a run to about 3.5 days instead of the usual 12–20 days. Hornung et al. [8] developed a novel reactor where both glucose and oxygen were fed directly to the BC-producing cells. These modified production processes were explored to increase the oxygen rich surface area to reactor volume ratio, which improves BC production.

An alternative method for BC production is submerged fermentation. Several strains of BC-producing bacteria had been screened for aerated and agitated cultivation [9,10]. Instead of a cellulose pellicle, small pellets of BC were produced. These BC pellets exhibit a lower degree of polymerization, crystallinity, and Young's modulus than that produced under static cultivation. The less-organized form of BC may have resulted from shear stress during agitation [11].

High biomass density also proved to be beneficial for BC production in many cases [12,13]. High density of biomass can be achieved by several ways such as cell immobilization, cell-recycle reactor, and hollow-fiber reactors. Cell-recycle reactors and hollow-fiber reactors have their limitations due to the high capital and operational cost, as well as the potential risk of membrane fouling and/or contamination during fermentation. Biofilm reactors, on the other hand, provide a substitute of high-biomass density systems with lower capital cost. Biofilm reactors have demonstrated a very high volumetric productivity of submerged fermentation, especially the continuous cultures when compared with suspended-cell cultures due to the high cell density maintained in the reactor [14].

Biofilms grow on the solid support when microorganisms attach, and are a natural form of cell immobilization [15]. Plastic composite support (PCS) is an extrusion product of a mixture between polyprolylene and nutritious compounds [16]. Polypropylene acts as a matrix and integrates agricultural mixtures, such as ground soybean hulls, soybean flour, and microbial nutrients such as bovine albumin, red blood cells, yeast extract, and peptone, as well as mineral salts. As a result, PCS not only provides an ideal physical structure for biofilm formation, but also releases nutrients slowly during fermentation.

Many studies showed that using PCS biofilm reactors can enhance production of ethanol, organic acid, and bacteriocin [14,17-26]. Therefore, a PCS biofilm reactor system was evaluated for BC production by *A. xylinum*, which is suitable for submerged fermentation [27].

The goal of this study was to evaluate effects of PCS implementation on bacterial cellulose production by *A. xylinum*, which was divided into two specific objectives: (1) to evaluate BC production in a PCS biofilm reactor and compare to one produced from the conventional suspended-cell culture, and (2) to analyze the material properties of the produced BC, including degree of crystallinity and crystal size by X-ray diffraction (XRD), thermogravimetric analysis (TGA) to determine its water content and thermal decomposition behavior, scanning electron microscopy analysis (SEM) for determining the structure of BC, and dynamic mechanical analysis (DMA) for determining tensile strength of BC.

## Materials and methods

### Microorganism

The bacterial strain used in this study was *Acetobacter xylinum *(ATCC 700178) obtained from the American Type Culture Collection (Rockville, MD), which was grown in CSL-Fru medium with agitation as a BC producer. The cell suspension of *A. xylinum *was stored at -80°C in a 20% glycerol solution. One milliliter of cell suspension stored at -80°C was added to 100 ml of CSL-Fru medium in a 500 ml flask and statically cultivated at 30°C for 3 days. The cellulose pellicle formed on the surface of the broth was homogenized using a homogenizer (model 7011S, Waring Co., Torrington, CT) at 10,000 rpm for 1 min and filtered through a sterile gauze to remove BC. Ten ml of the filtrate containing the cell suspension was added to 90 ml of CSL-Fru medium and cultivated at 30°C and 200 rpm for 24 h and used as an inoculum.

### Media

All the chemicals used were of analytical grade and commercially available unless specified description. For BC production, corn steep liquor with fructose (CSL-Fru) medium was slightly modified as previously described [27], and contained the following constituents per liter: 50 g of fructose, 20 ml of CSL (corn steep liquor, Nihon Starch Industry, Kagoshima, Japan), 1.0 g of KH_2_PO_4_, 0.25 g of MgSO_4_. 7H_2_O, 3.3 g of (NH)_2_SO_4_, 3.6 mg of FeSO_4_. 7H_2_O, 1.5 mg of CaC1_2_. 2H_2_O, 2.4 mg of Na_2_MoO_2_. 2H_2_O, 1.7 mg of ZnSO_2_. 7H_2_O, 1.4 mg of MnSO_4_. 5H_2_O, 0.05 mg of CuSO_4_. 5H_2_O, 2.0 mg of inositol, 0.4 mg of nicotinic acid, 0.4 mg of pyridoxine. HCl, 0.4 mg of thiamine. HCl, 0.2 mg of pantothenic acid Ca salt, 0.2 mg of riboflavin, 0.2 mg of pamino-benzoic acid, 0.002 mg of folic acid, and 0.002 mg of biotin. Agar (Becton, Dickinson Co., Sparks, MD), carboxymethylcellulose (Fluka Co., Buchs SG, Switzerland), microcrystalline cellulose (FMC Co., Newark, DE), and sodium alginate (Sigma, St. Louis, MO) were used as additives at various concentrations.

### Plastic composite support

Thirteen types of PCS tubes (Table 1) were manufactured in the Center for Crops Utilization Research (CCUR) at Iowa State University using a twin-screw corotating Brabender PL2000 extruder (model CTSE-V; C.W. Brabender Instruments, Inc., South Hackensack, NJ) as described by Ho et al. [24]. Polypropylene and other ingredients of PCS were mixed together before being extruded at 13 rpm through a medium pipe die with barrel temperatures of 200, 220, and 200°C and a die temperature of 165°C. To randomize manufacturing effects, 500 g of the mixture was extruded twice for each blend in a random order to ensure reproducibility, producing 1 kg of PCS from each blend. The resulting tubes had a wall thickness of 2.5 mm and an outer diameter of 10.5 mm. For bioreactor trials, these PCS tubes were cut into 6.0-cm length with both ends cut at a 45° angle to allow better flow of medium inside the tubes. For testing and selection of PCS in test tube fermentations, the PCS tubes were cut into small disks with a thickness of 3 mm using a band saw.

**Table 1:** List of PCS ingredients

**Support**	**% dry weight**						
	
	**PP^a^**	**S^b^**	**D^c^**	**F^d^**	**Y^e^**	**R^f^**	**MS^g^**
S	50	50					
SB+	50	45	5				+
SF+	50	45		5			+
SFB	50	40	5	5			
SFR	50	40		5		5	
SFY	50	40		5	5		
SFYB+	50	35	5	5	5		+
SFYR+	50	35		5	5	5	+
SR+	50	45				5	+
SY+	50	45			5		+
SYB+	50	40	5		5		+
SFYBR	50	30	5	5	5	5	
SFYB	50	35	5	5	5		

^a ^PP, polypropylene; Quantum USI Division, Cincinnati, OH. ^b ^S, dried ground (20-mesh) soybean hulls; Cargill Soy Processing Plant, Iowa Falls, IA. ^c ^B, dried bovine albumin; Proliant Corp., Ames, IA. ^d ^F, defatted soy bean flour; Archer Daniels Midland, Decatur, IL. ^e ^Y, yeast extract ardamine Z; Red Star Bioproducts, Juneau, WI. ^f ^R, dried bovine red blood cell; Proliant Corp., Ames, IA. ^g ^MS, mineral salts; per kilogram of PCS blend, 2.72 g of sodium acetate, 0.004 g of MgCl2â6H2O, and 0.02 g of NaCl.

### Flask Fermentations for Selection of PCS

Thirteen types of PCS with different compositions were evaluated for both biofilm formation and BC production using flask fermentation with three replicates. For each replicate, 3 g of PCS disks was sterilized dry in 150 mL flask for 1 h at 121°C. Fifty mL of sterilized medium with 5% (w/v) fructose as a carbon source was added aseptically to the sterile PCS before being incubated at 30°C overnight in order to hydrate the PCS. The medium was then aseptically decanted, and fresh sterile medium was added with 1% (v/v) inoculum of 24-h *A. xylinum *before incubating at 30°C for 120 h. Each replicate was determined the biomass on the PCS, BC production, and residual fructose. The best PCS type was selected according to the biofilm formation on PCS (g-biomass/g-PCS), BC production (g/mL), nitrogen content (% w/w), and nitrogen leaching rate from PCS (% w/w of total nitrogen after the 120-h fermentation). A control experiment without PCS rings was also performed simultaneously. The results on nitrogen content and nitrogen leaching rate were obtained from the study of Ho et al. [22].

### BC Fermentation in the Bioreactor

**Figure 1:**
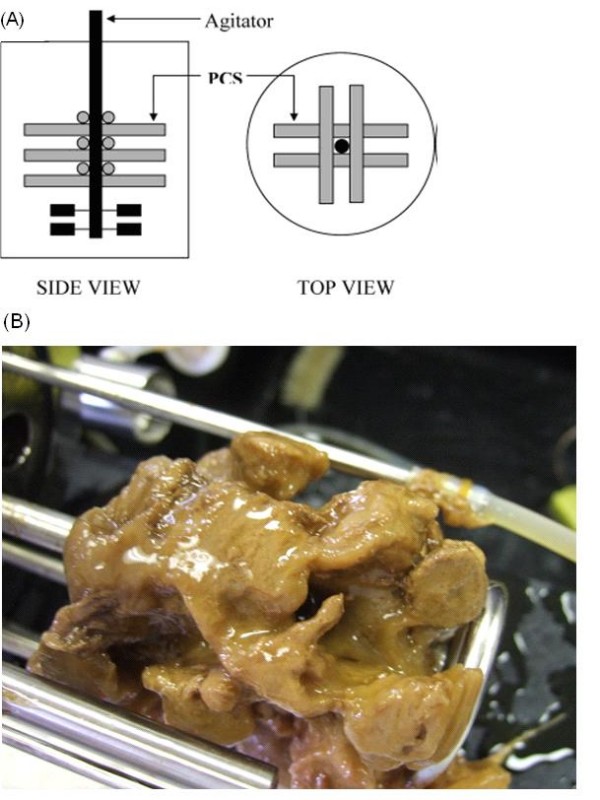
**(A) Diagram of the PCS biofilm reactor, and (B) Cellulose grown on the PCS shaft after 120 hr cultivation**.

BC fermentations were conducted in a 1.25-L Bioflo 3000 fermentor (New Brunswick Scientific, Edison, NJ) at 30°C with a working volume of 1 L and the agitation rate of 100 rpm. For the biofilm reactor, 12 PCS tubes were bound to the agitator shaft in a gridlike fashion, with 6 rows of two parallel tubes (Figure 1). The reactor vessel with PCS was autoclaved with water at 121°C for 45 min. Fructose and nitrogenous components with mineral salts were autoclaved separately and added to the reactor aseptically after draining the water from the reactor as recommended by Ho et al. [24]. After inoculation with a 24-h culture of *A. xylinum *(10% v/v), 120-h batch fermentation was carried out. The initial pH was set at 5.0, but pH was not controlled during fermentation. After each run, the biomass on the PCS, BC production, and residual fructose were evaluated. Suspended-cell fermentation as control (i.e. without PCS) was conducted using the same protocol except that the reactor containing medium without PCS was autoclaved and inoculated with 24-h culture of *A. xylinum *(10% v/v) at the beginning of each batch. Each treatment was duplicated.

### Measurement of biomass and bacterial cellulose

At the end of the 5-day incubation, BC on the PCS shaft was detached using a knife and combined with culture broth and then centrifuged at 3300 × *g *for 20 min (Super T-21, Sorvall Co., Norwich, CT). For biomass, the precipitated pellicle was added to 90 ml of buffer (0.1 M potassium acetate-acetate buffer (pH 5.0) and 10 ml of 20% cellulase (Celluclast, Novo Nordisk A/S, Denmark) and incubated at 50°C with shaking at 100 rpm for 1 h to hydrolyze BC [27]. Then, the solution was centrifuged at 3,300 × *g *for 20 min. The precipitate was dried in an oven at 80°C overnight and then weighed to determine biomass. For BC determination, the precipitated BC pellicle was treated with 0.1 N NaOH solution at 80°C for 30 min to remove the bacterial cells and medium components [28]. This NaOH treatment was repeated three times and then, the solution was centrifuged at 3,300 × *g *for 20 min. The obtained cellulose was dried in an oven at 80°C overnight and then weighed.

### Fructose

Fructose concentration was determined using a Waters high-performance liquid chromatography unit equipped with column heater, autosampler, computer controller, and a refractive index detector (Waters, Franklin, MA). Components were separated on a Bio-Rad Aminex HPX-87H column (300 mm × 7.8 mm) (Bio-Rad, Richmond, CA) with 0.012 N sulfuric acid as a mobile phase at a flow rate of 0.8 ml/min with an injection volume of 20 μl and a column temperature of 65°C. Before injection, the samples were centrifuged in a microcentrifuge at 2,000 × *g *for 5 min (Model C-1200, National Labnet Co., Woodbridge, NJ) and filtered through 0.22 μm filters (13 mm diameter disk filters, Millipore, Bedford, MA) to remove suspended solid particles.

### X-ray diffraction

To determine the crystallinity of the produced BC, the X-ray diffraction (XRD) patterns of the samples were collected on a Scintag PAD V theta-2-theta diffractometer (Scintag, Cupertino, CA) using a copper x-ray source. Scans were collected at 2 deg per minute from 5–70 degree 2θ. Samples of BC were lyophilized overnight at 0.133 mbar first by using a lab-scale freeze dryer (Model Freezoom 2.5 L, Labconco co., Kansas, MO) and pressed into a thin and flat layer (~1.0 mm thickness) for analysis. MDI Jade 8 software (Materials Data, Inc., Livermore, CA) was used to process the diffraction pattern and to calculate the crystallinity of BC. The degree of crystallinity was taken as Cr I = (I_200 _- I_am_)/I_200_, where I_200 _is the overall intensity of the peak at 2θ about 22.9° and I_am _is the intensity of the baseline at 2θ about 18° [29]. The crystal size was calculated by the Scherrer equation [30]:



where *β *is the breadth of the peak of a specific phase (hkl), *K *is a constant that varies with the method of taking the breadth (0.89<*K*<1), *λ *is the wavelength of incident x-rays, *θ *is the center angle of the peak, and *L *is the crystallite length (size).

### Field emission scanning electron microscopy (FESEM)

Both BC samples before and after removal of cells were lyophilized overnight at 0.133 mbar and then coated with thin Platinum film around 5 nm. A LEO 1530 field emission scanning electron microscope (Leo Co., Oberkochen, German) operating at 2 kV was used for examination of the samples. An imaging magnification of approximately 27,000, 7,000, and 250 times was used for BC, bacteria, and PCS rings, respectively.

### Thermogravimetric analysis (TGA)

The dynamic weight loss tests were conducted on a thermogravimetric analyzer (TGA) machine (model Q500, TA instruments-Water LLC, New Castle, DE). For water content determination of cellulose, all sample tests were conducted in a N_2 _purge (40 ml/min) over a temperature range 30–650°C at an increase rate of 10°C/min. BC samples were placed on the absorbent wipers to eliminate excess water. For thermal decomposition behavior test, cellulose samples were dried at 80°C and tests were then conducted in a N_2 _purge (40 ml/min) over a temperature range 80–650°C at an increase rate of 10°C/min. The initial weight at 80°C was set as 100%.

### Mechanical properties of bacterial cellulose

The mechanical properties of BC were performed by a dynamic mechanic analyzer (DMA) (model Q800, TA instrument-Water LLC, New Castle, DE). BC from agitation cultivation after removal of cells was lyophilized first and pressed into a flat piece at 2,500 psi using a press. The BC samples were then cut to make 20 × 5 mm plates for testing. Sample were mounted between upper (fix) and lower (movable) clamps, and two ends were fixed to avoid slip. Force was applied to the lower clamp to pull the sample in tension. Experiments were run at 0.1 N/min force. Tests were performed at an environmental temperature of 35°C. Stress (σ) was calculated by *F/A *where *A *is the area measured as width × thickness of sample and *F *is force in Newtons (N). Strain (ε) was calculated by *ΔL/L*_*o *_where *ΔL *is exerted extension from starting point *L*_*o*_. Young's modulus was calculated by Stress/Strain in the linear region.

### Statistical analysis

All treatments were replicated at least three times. The significant difference of the results was evaluated using the Generalized Linear Model (GLM; with p < 0.05) and Tukey's honestly significant differences (HSD) multiple comparison module of the MINITAB Statistical Software package (Release 13.30; State College, PA, USA)

## Results and Discussion

### PCS selection

**Figure 2:**
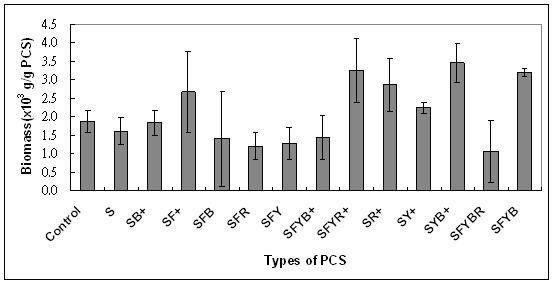
**Effects of different PCS blends on the weight of bacteria biomass on the PCS in test tube systems without pH control after 120 h (n = 3)**. (S, soybean hulls; F, soybean flour; Y, yeast extract; R, drued bovine RBC; B, dried bovine albumin; +, mineral salts).

**Figure 3:**
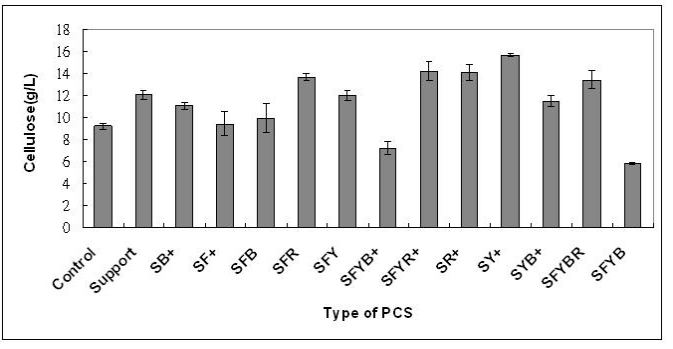
**Effects of different PCS blends on BC production in test tube systems without pH control after 120 h (n = 3)**. (S, soybean hulls; F, soybean flour; Y, yeast extract; R, drued bovine RBC; B, dried bovine albumin; +, mineral salts).

BC production (g/l) and biomass formation (g/g-PCS) in flask fermentation from three replicates of each PCS types are presented in Figures 2 and 3. When biomass vs. BC was plotted (not shown), the result showed no specific relationship between biomass attached on PCS and BC production as the correlation coefficients between these values were extremely low: R-squared values of 0.01 for biomass versus BC production. This pattern was also observed in the PCS biofilm fermentation of succinic acid and nisin [24,26]. Comparable levels of biomass were attached on each PCS, ranging from 1.1 to 3.3 g/g-PCS. In an attempt to select an optimal PCS for BC production, the means of BC produced (g/l) were ranked as follow according to the results from Tukey's multiple comparisons: SY+ > SFR, SFYR+, SR+, SFYBR > S, SB+, SFY, SYB+ > SFYB+ > SFYB and the ranking of biomass produced (g/g) on PCS was: SFYR+, SYB+, SFYB > SF+, SR+, SY+ > S, SB+, SFB, SFR, SFY, SFYB+, SFYBR. Overall, after considering other factors such as nitrogen content per g of PCS, nitrogen leaching rate, and amounts of biomass attached on PCS, SFYR+ was selected as solid support for BC production by *A. xylinum *in a batch biofilm reactor due to its high nitrogen content, moderate nitrogen leaching rate [22-24], and high biomass attachment (Figure 2).

### BC production by *A. xylinum *in the PCS biofilm reactor

*A. xylinum *was grown in CSL-Fru medium with the selected PCS in a biofilm reactor. Fermentation in the PCS biofilm reactor yielded 7.05 g/l BC, which is 2.5-fold greater than BC production in the suspended-cell reactor (2.82 g/l). This result was probably due to the higher biomass density accumulated on PCS and the cell-density-dependent BC production relation as previously described [9,12].

### BC structure and physical properties

**Figure 4:**
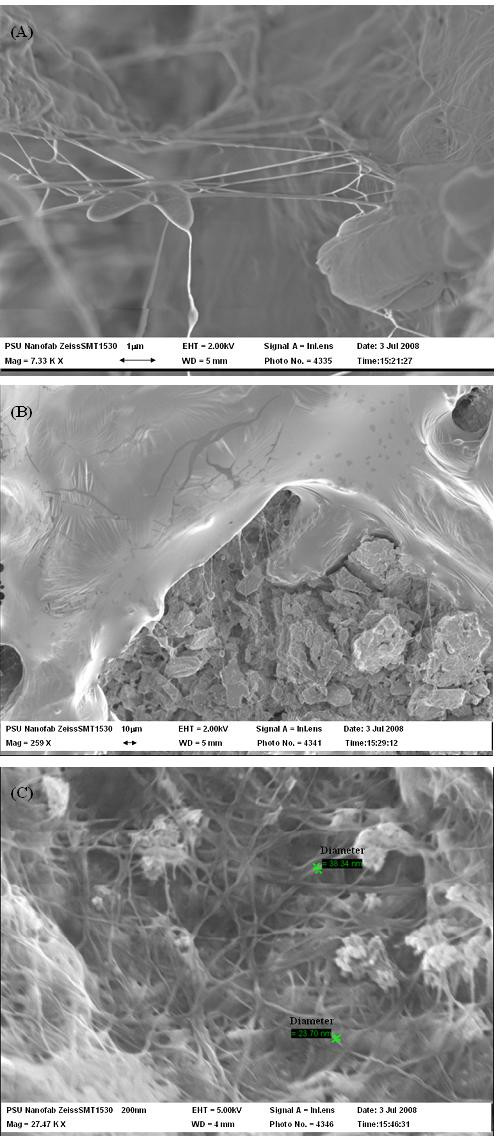
**Visualization of FESEM pictures of PCS-grown BC after freeze dried**. (A) *A. xylinum *attached on PCS and produced BC (The arrow here indicates *A. xylinum*), (B) BC on the PCS, and (C) BC structure produced by *A. xylinum *on PCS.

Instead of forming small chunks within cultivation broth, BC produced in the PCS biofilm reactor attached on the PCS shaft. Figure 1(B) shows the phenomenon of BC produced on the PCS due to the accumulation of biomass on it. The morphological structures of BC fibers from lyophilized BC samples with and without removal of cells were analyzed by FESEM. Figure 4(B) shows that *A. xylinum *as indicated with an arrow in the figure are producing an extracellular cellulose, where as twisting ribbon of cellulose and forming BC on PCS surface is shown in Figure 4(B). PCS-grown BC detached from PCS after removal of cells was also analyzed. Figure 4(C) showed that the BC sample retained its interweaving structure of fibers. The width of BC fibers had a broader range than control, around 15~40 nm, which could influence BC properties such as water-holding capacity, thermal stability, and mechanical strength.

**Figure 5:**
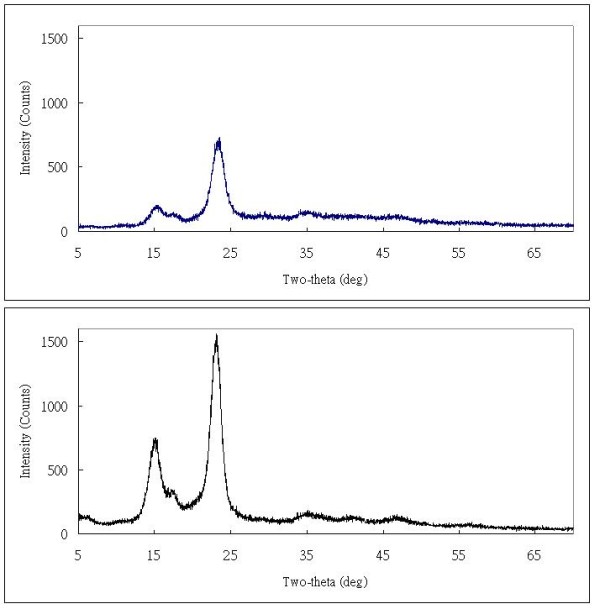
**X-ray diffraction patterns of bacterial cellulose produced by *A*. *xylinum *in a PCS biofilm reactor (A), and suspended-cell reactor (B)**.

XRD patterns obtained from our BC samples demonstrated three main characteristic peaks standing for crystal plane <-110>, <110>, and <200> (Figure 5), which showed that the cellulose exhibited primarily the I-β pattern [31]. The crystalline index of PCS-grown BC was higher than BC harvested from the agitated reactor (Table 2). The crystallinity of BC increased slightly from 85% to 93% when PCS was used as a solid support in the medium, which may arise as a result of longer polymerization of BC when compared to control BC from suspended-cell fermentation. The crystal size of <200> crystal plane remained the same around 5.2 nm.

**Table 2:** Comparison of crystallinity and crystal size between BC from PCS biofilm and suspended-cell reactor.

**BC types**	**Crystallinity** **(%)**	**Crystallite size of** **<200> (nm)**
PCS-grown BC	93 ± 2.1	5.2 ± 0.3
Control	85 ± 1.5	5.2 ± 0.5

### Thermogravimetric analysis

**Figure 6:**
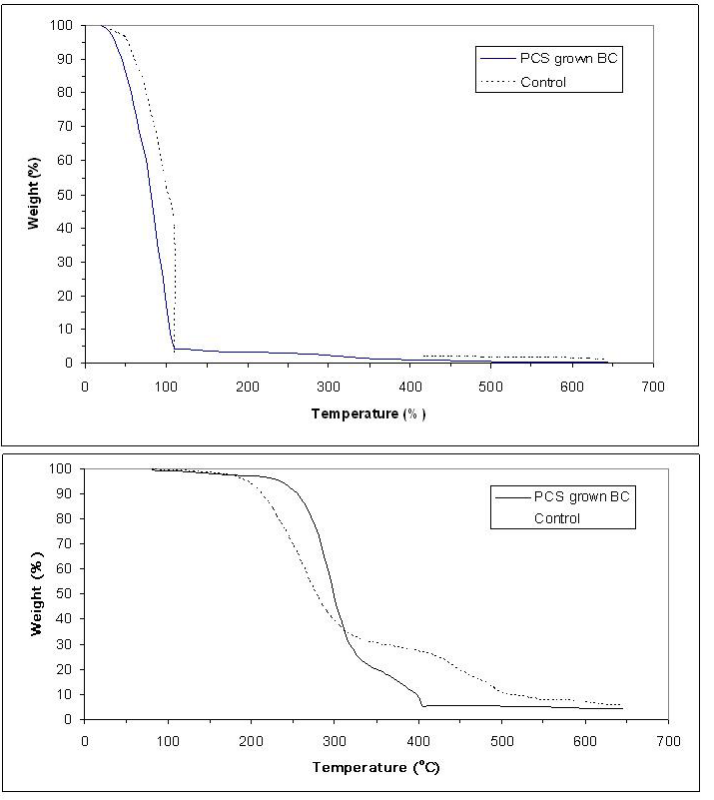
**The TGA curves of BC samples produced on biofilm and suspended-cell reactor before (A) and after (B) removal of free water**.

**Figure 7:**
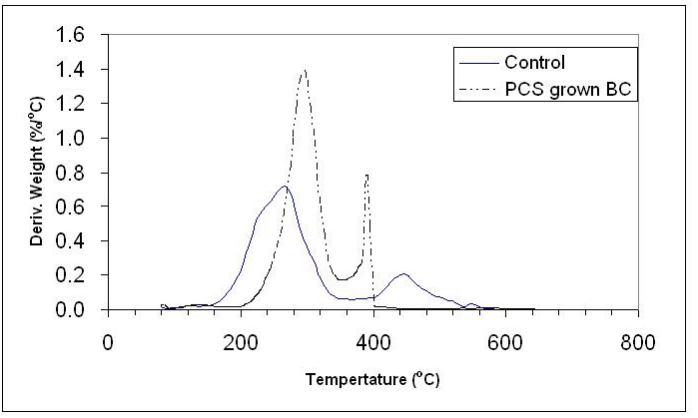
**Derivative TGA patterns of BC from PCS biofilm and suspended-cell reactor**.

To determine water retention and information on thermal decomposition behavior of PCS-grown BC, thermogravimetric analysis (TGA) was performed on BC samples at the end of cultivation with and without PCS present. The TGA thermograms of BC samples are shown in Figure 6. PCS-grown BC exhibited around 95% water retention ability, which is lower than BC from the suspended-cell reactor (98%) (Figure 6A), but higher than a BC pellicle from static culture (73%) as reported earlier by Seifert et al. [32]. The results are probably due to incorporation of some water-insoluble particles of PCS, that alter the morphology of the BC structure (Figure 4C). For thermal decomposition behavior of BC samples, dried BC samples were used. Figure 6B showed two distinct steps for weight loss of BC samples, indicating the possibility of two types of decomposition. Yang and Chen [33] illustrated that the initial weight loss at lower temperature ranging from 200°C to 360°C is attributed to the removal of small molecular fragments such as hydroxyl and methylhydroxyl groups. The second weight loss ranging from 360°C to 600°C showed the degradation of polymeric chains and the six-member cyclic structure, pyran. Since the thermal degradation behavior is affected by some structure parameters such as molecular weight, crystallinity, and orientation [34], the more sharp decrease in weight of PCS-grown BC at both stages could be due to its higher crystallinity, degree of polymerization and compact interweaving structure. The maximum decomposition temperature (*T*_max_), known as a criterion of thermal decomposition position, was calculated from different TGA curves. Each peak on the *T*_max _pattern represents the steepest slope of weight loss (%/°C) for each step during decomposition. The PCS-grown BC displayed two peaks (Figure 7), one at 265°C and another at 445°C. In the cases of PCS-grown BC, the *T*_max _of first peak increased by about 30°C, which indicated PCS-grown BC possessed higher thermal stability and began to degrade at higher temperature.

### Mechanical properties

**Figure 8:**
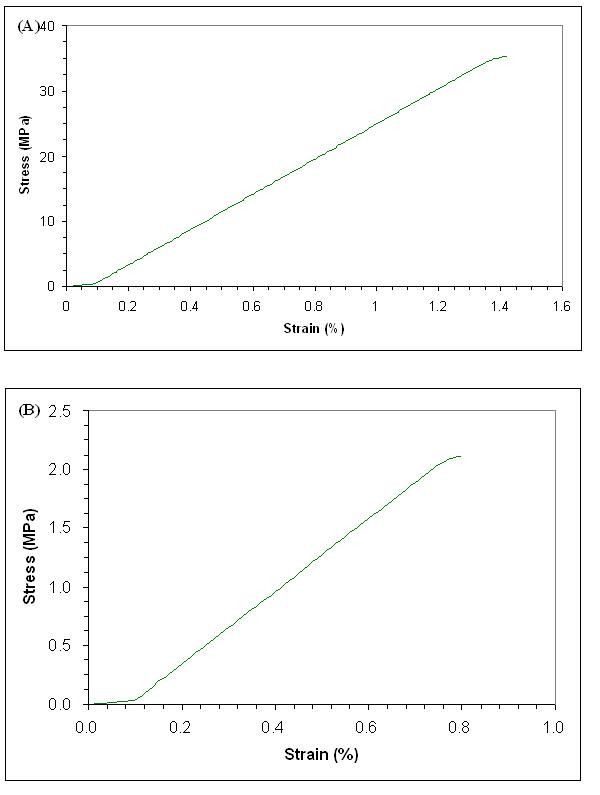
**Stress/strain diagrams of tensile test results**. (A) PCS-grown BC. (B) Control BC.

BC produced in either agitated or air-lifted culture is believed to lose its high polymerization property which results in low mechanical strength [35] due to the formation of small pellets. As a result, DMA analysis was performed to determine if there was any improvement in mechanical strength of PCS-grown BC as compared to control BC. Both types of BC were investigated: PCS-grown BC from a PCS biofilm reactor, and control from agitated culture. Figure 8 shows a typical stress-strain response of the PCS-grown BC (A) and control (B) samples. The extension of our testers showed a linear-elastic behavior. PCS-grown BC exhibited a longer elastic deformation around 1.4%, where a yield point was seen, which was followed by irreversible deformation. On the other hand, control BC testers showed only 0.8% elongation.

**Figure 9:**
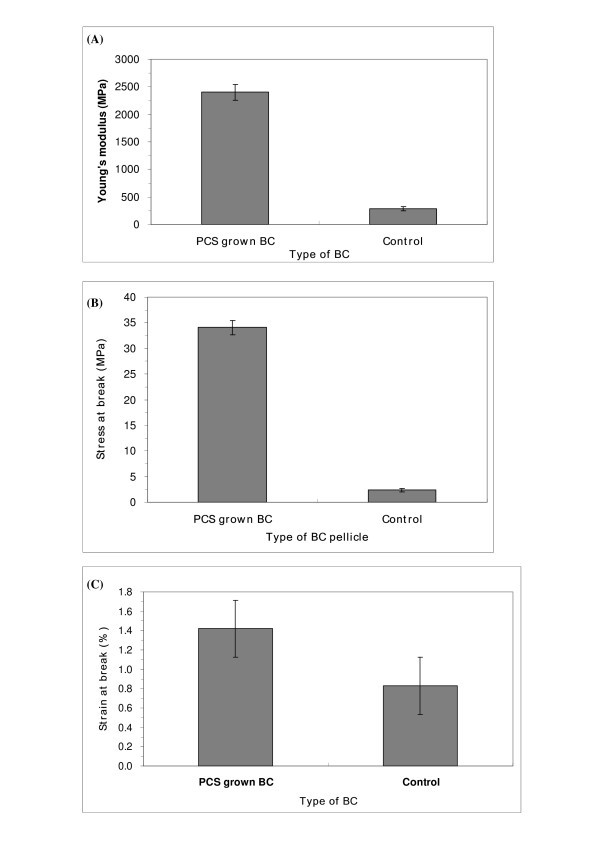
**Results of tensile test of BC**. (A) Stress at break; (B) Strain at break; (C) Young's modulus (n = 5).

Figure 9 shows the mean value (μ) and standard deviation (*σ*) of the tested samples' tensile properties (n = 5). Tensile strength (*σ*_max_), deformation at break (*ε*_max _(%)) and Young's modulus (*E *(MPa)) were measured. BC tested from the PCS biofilm reactor showed a significant increase in both stress at break and Young's modulus when compared to BC from agitated culture. The stress at break increased from 2.8 to 34.2 MPa (PCS-grown BC). Young's modulus increased from 286 to 2,401 MPa (PCS-grown BC). The strain at break also increased from 0.8 to 1.4%. The Young's modulus showed that mechanical strength of PCS-grown BC increased 840% when compared to control BC. We hypothesize that this improvement is due to an increase in glucose polymerization of the cellulose produced, or due to the incorporation of PCS components.

## Conclusion

Based on the results of this study, BC production by *A. xylinum *can be enhanced using a PCS biofilm reactor system. The material properties of BC are changed when grown in the presence of the PCS. BC produced in a biofilm reactor grew primarily on the PCS supports and is mainly in cellulose I-β pattern. Among thirteen types of PCS, PCS blended with dried soybean hulls, defatted soybean flour, yeast extract, dried bovine red blood cells, and mineral salts (SFYR+) was selected for biofilm BC fermentation. The PCS biofilm reactor yielded the highest BC (7.05 g/l) which is 2.5 fold compared to control (2.82 g/l). The XRD studies indicated that crystallinity of PCS-grown BC is about 93%, which is higher than control, and a crystal size of 5.2 nm in <200> crystal plane. FESEM image provided the evidence that *A. xylinum *can grow and produce BC on the PCS. TGA results showed that PCS-grown BC possessed 95% water content and demonstrated higher thermal stability (~30°C) when compared to control BC. BC harvested from the biofilm reactor also exhibited higher mechanical strength than BC produced from agitated culture. The PCS-grown BC exhibited higher stress at break, Young's modulus, and strain at break than one produced from agitated culture.

In conclusion, PCS biofilm reactor system successfully demonstrated several advantages for BC production, such as enhanced production yield, higher thermal stability, and stronger mechanical strength. Based on these results, the development of a semi-continuous and continuous fermentation process for producing BC will be the next challenge. Further study on determining other properties of BC produced using PCS, such as gas-penetration ability, metal-ion chelating capacity, and crystallization characteristics are needed.

## Competing interests

The authors declare that they have no competing interests.

## Authors' contributions

KC carried out the whole experiment including fermentation and material property analysis. JC conceived of the study, and participated its design and coordination. AD conceived of the study, and participated its design and coordination. All authors read and approved the final manuscript.
